# Supplementary material for: More Than a Decade of GeneXpert® Mycobacterium tuberculosis/Rifampicin (Ultra) Testing in South Africa: Laboratory Insights from Twenty-Three Million Tests
Source: Diagnostics (Basel). 2023 Oct 19;13(20):3253. doi: 10.3390/diagnostics13203253 (PMC10605857; doi:10.3390/diagnostics13203253)
Supplement: Supplementary file 1 [file diagnostics-13-03253-s001.zip › diagnostics-2561737-supplementary.pdf]

Table S1. Analysis of Xpert MTB/RIF Ultra tested volumes and *Mycobacterium tuberculosis* complex Trace detected results by province.

Province	Tested Volumes Xpert MTB/RIF Ultra n= (%)	MTBC Trace Detected n= (%)
Eastern Cape	1,939,442 (17.0)	42,157 (2.2)
Free State	416,970 (3.6)	9034 (2.2)
Gauteng	1,744,726 (15.3)	25,766 (1.5)
KwaZulu-Natal	3,928,929 (34.4)	44,241 (1.1)
Limpopo	645,548 (5.7)	7502 (1.2)
Mpumalanga	582,265 (5.1)	11,326 (2.0)
North West	563,068 (4.9)	12,106 (2.2)
Northern Cape	324,160 (2.8)	7029 (2.2)
Western Cape	1,280,149 (11.2)	32,319 (2.5)
Total	11,425,257 (100.0)	191,480 (1.7)

MTBC: *Mycobacterium tuberculosis* complex.

Table S2. *Mycobacterium tuberculosis* complex trace detected rate by specimen type for Xpert MTB/RIF Ultra tested specimens.

Specimen Type	Specimens Tested n= (%)	MTBC Trace Detected n= (%)
Unknown origin	391,375 (3.4)	6289 (1.6)
Specimens of pulmonary origin		
Bronchial brushings	5705 (0.0)	291 (5.1)
Gastric aspirate	124,145 (1.1)	2172 (1.7)
Nasopharyngeal	515 (0.0)	6 (1.2)
Sputum	10,377,022 (90.8)	168,214 (1.6)
Tracheal aspirate	20,264 (0.2)	515 (2.5)
Specimens of extra-pulmonary origin		
Aspirate/FNA	7735 (0.1)	335 (4.3)
CSF	273,228 (2.4)	4714 (1.7)
Fluid	162,419 (1.4)	7608 (4.7)
Pus/abscess	30,769 (0.3)	374 (1.2)
Stool	299 (0.0)	4 (1.3)
Tissue	18,945 (0.2)	622 (3.3)
Urine	12,406 (0.1)	336 (2.7)
Total	11,425,257 (100.0)	191,480 (1.7)

MTBC: *Mycobacterium tuberculosis* complex.

Table S3. Distribution of *Mycobacterium tuberculosis* complex detection rate in cerebrospinal fluid by age category.

Age in years	CSF Tested n= (%)	MTBC Detected n= (%)
0-1	40,023 (10.8)	876 (2.2)
2-3	7490 (2.0)	189 (2.8)
4-5	4051 (1.1)	112 (3.4)
6-7	3032 (0.8)	77 (2.5)
8-10	3899 (1.1)	87 (4.7)
11-14	5661 (1.5)	157 (4.2)
15-25	43,836 (11.9)	1488 (2.8)
26-35	85,763 (23.2)	4030 (3.2)

36-45	79,100 (21.4)	3339 (2.3)
46-55	46,081 (12.5)	1465 (2.5)
56-65	26,160 (7.1)	609 (2.2)
>65	16,592 (4.5)	255 (1.5)
Unknown	7563 (2.0)	185 (2.4)
Total	369,251 (100.0)	12,869 (3.5)

CSF: cerebrospinal fluid; MTBC: *Mycobacterium tuberculosis* complex.

Table S4. Analysis of testing by age category and gender for GeneXpert testing by specimens tested, *Mycobacterium tuberculosis* complex and rifampicin resistance detection rates.

**Category**	**Specimens Tested n= (%)**	**MTBC Detected n= (%)**	**RIF Resistance Detected n= (%)**
		Age in years	
0-1	600,595 (2.5)	31,164 (5.2)	2788 (8.9)
2-3	145,287 (0.6)	4833 (3.3)	221 (4.6)
4-5	170,409 (0.7)	3892 (2.3)	183 (4.7)
6-7	268,292 (1.1)	3538 (1.3)	223 (6.3)
8-10	466,673 (2.0)	7553 (1.6)	566 (7.5)
11-14	720,884 (3.0)	21,617 (3.0)	1319 (6.1)
15-25	3,560,745 (15.0)	353,176 (9.9)	18,339 (5.2)
26-35	5,325,637 (22.4)	659,818 (12.4)	40,468 (6.1)
36-45	4,546,467 (19.2)	539,812 (11.9)	34,268 (6.3)
46-55	3,426,771 (14.4)	310,063 (9.0)	17,429 (5.6)
56-65	2,352,375 (9.9)	157,658 (6.7)	7348 (4.7)
>65	1,642,512 (6.9)	83,666 (5.1)	3212 (3.8)
Unknown	514,021 (2.2)	51,687 (10.1)	4089 (7.9)
		Gender	
Female	11,997,695 (50.5)	801,775 (6.7)	51,715 (6.5)
Male	10,165,069 (42.8)	1,221,524 (12.0)	65,654 (5.4)
Unknown	1,577,904 (6.6)	205,178 (13.0)	13,084 (6.4)

MTBC: *Mycobacterium tuberculosis* complex; RIF: rifampicin.
